# Supplementary material for: Optimizing mouse metatranscriptome profiling by selective removal of redundant nucleic acid sequences
Source: mSystems. 2025 Jun 16;10(7):e00167-25. doi: 10.1128/msystems.00167-25 (PMC12282169; doi:10.1128/msystems.00167-25)
Supplement: Supplemental material — Supplemental figures and tables. [file msystems.00167-25-s0002.pdf]

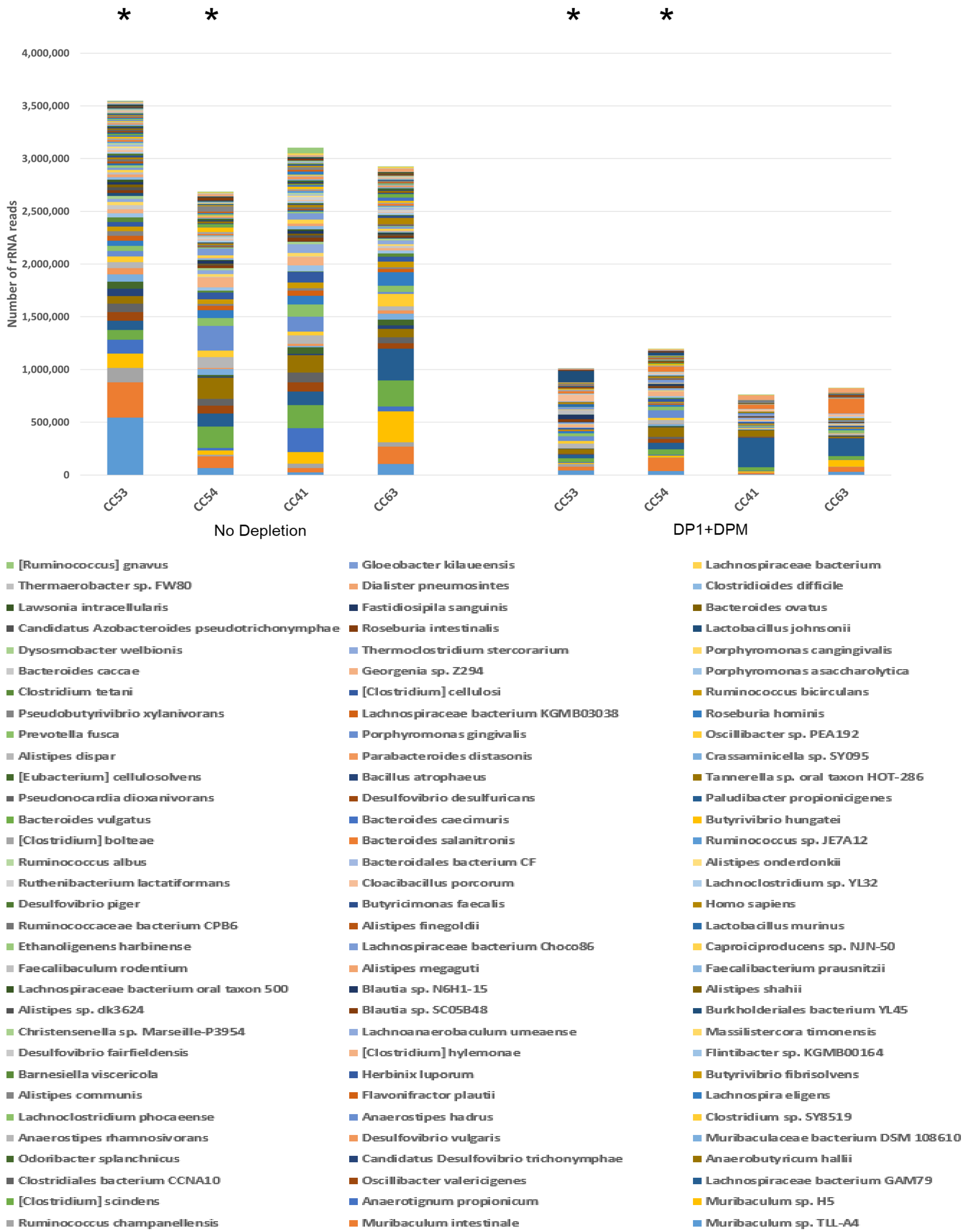


**Supplementary Figure 1:** Taxonomic analysis of the collected rRNA reads from the top 100 most abundant species (sorted by sample CC53 – No Depletion) either from undepleted (left side) or samples depleted with DP1 & DPM (right side) indicating that no particular species dominate the remaining rRNA content of the samples following depletion. See Figure 1C for the Top 15 species. Samples CC54, CC53, CC41, CC63. The asterisks at the top indicate two of the samples chosen for probe design in Figure 2.

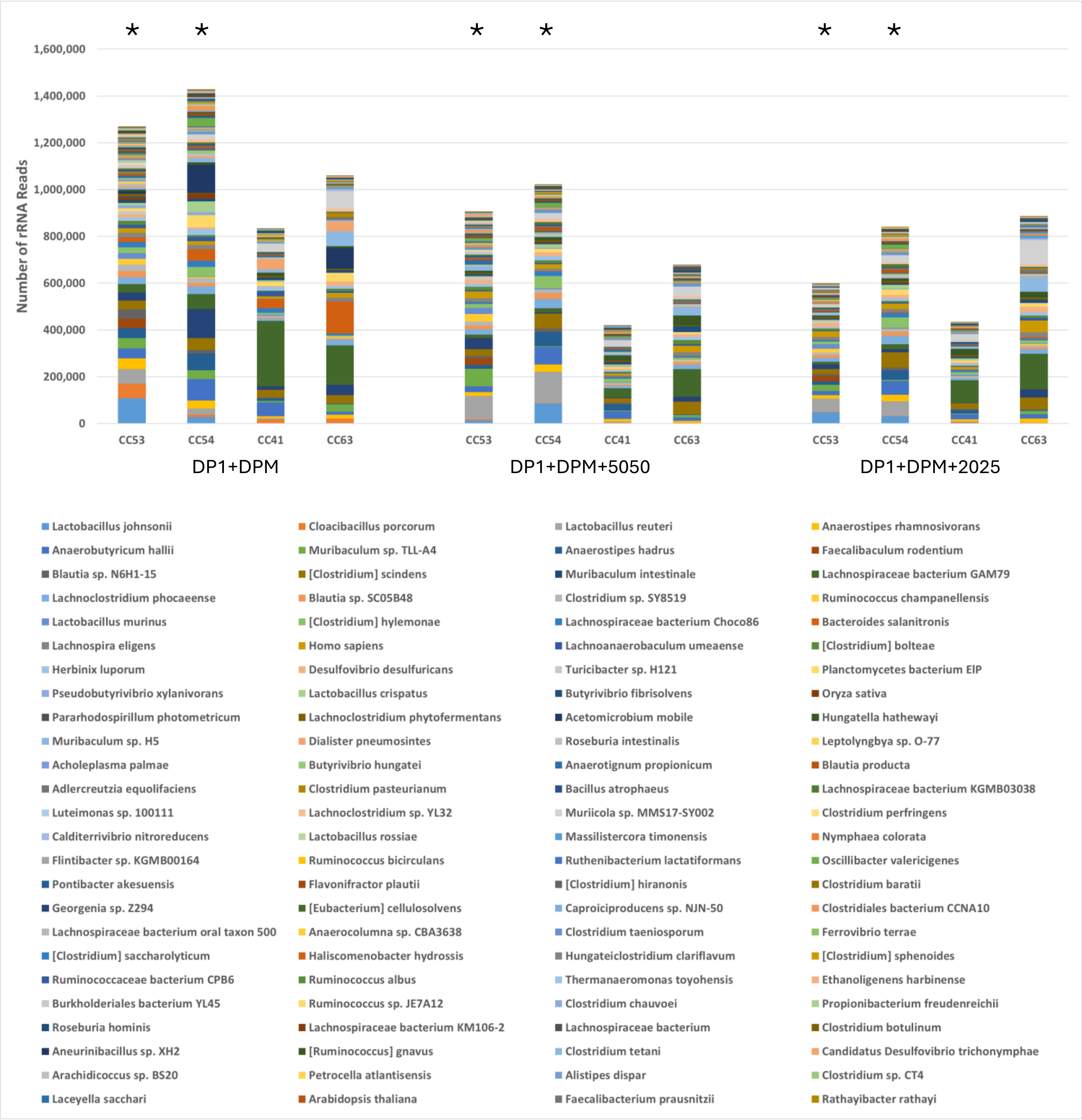


**Supplementary Figure 2:** Taxonomic analysis of the collected rRNA reads from the top 100 most abundant species (sorted by sample CC53 – DP1+DPM) either from samples depleted with DP1+DPM (left side), DP1+DPM+5050 (middle), or DP1+DPM+2025 (right side) indicating that no particular species dominate the remaining rRNA content of the samples following depletion. See Figure 3C for the Top 15 species. The asterisks at the top indicate two of the samples chosen for probe design in Figure 2.

| Mouse Name – Cecum | Mouse Name – Ileum | Mouse Name – Liver | Mouse Lab ID |
|---|---|---|---|
| N/A | IL1 | LVR1 | 101 |
| CC2 | IL2 | LVR2 | 102 |
| CC3 | IL3 | LVR3 | 104 |
| CC4 | IL4 | LVR4 | 105 |
| CC5 | IL5 | LVR5 | 106 |
| CC6 | IL6 | LVR6 | 107 |
| CC7 | IL7 | LVR7 | 108 |
| CC8 | IL8 | LVR8 | 109 |
| CC9 | IL9 | LVR9 | 114 |
| N/A | IL10 | LVR10 | 115 |
| CC11 | IL11 | LVR11 | 116 |
| CC12 | IL12 | LVR12 | 117 |
| N/A | IL13 | N/A | 118 |
| CC14 | IL14 | LVR14 | 119 |
| CC15 | IL15 | LVR15 | 120 |
| N/A | IL16 | LVR16 | 121 |
| CC17 | IL17 | LVR17 | 122 |
| CC18 | IL18 | LVR18 | 124 |
| CC19 | IL19 | LVR19 | 125 |
| CC20 | IL20 | LVR20 | 126 |
| N/A | IL21 | LVR21 | 127 |
| CC22 | IL22 | LVR22 | 128 |
| CC23 | IL23 | LVR23 | 129 |
| CC24 | IL24 | LVR24 | 131 |
| CC25 | IL25 | LVR25 | 132 |
| N/A | IL26 | LVR26 | 134 |
| CC27 | IL27 | LVR27 | 135 |
| CC28 | IL28 | LVR28 | 136 |
| CC29 | IL29 | LVR29 | 142 |
| CC30 | IL30 | LVR30 | 144 |
| CC31 | IL31 | LVR31 | 145 |
| CC32 | IL32 | LVR32 | 201 |
| CC33 | IL33 | LVR33 | 202 |
| CC34 | IL34 | LVR34 | 203 |
| CC35 | IL35 | LVR35 | 204 |
| CC36 | IL36 | LVR36 | 205 |
| CC37 | IL37 | LVR37 | 206 |
| CC38 | IL38 | LVR38 | 207 |
| CC39 | IL39 | LVR39 | 208 |
| CC40 | IL40 | LVR40 | 210 |
| CC41 | IL41 | LVR41 | 211 |
| CC42 | IL42 | LVR42 | 212 |
| CC43 | IL43 | LVR43 | 216 |
| N/A | IL44 | LVR44 | 217 |
| CC45 | IL45 | LVR45 | 218 |
| CC46 | IL46 | LVR46 | 219 |
| CC47 | IL47 | LVR47 | 220 |
| CC48 | IL48 | LVR48 | 222 |
| CC49 | IL49 | LVR49 | 223 |
| CC50 | IL50 | LVR50 | 230 |
| CC51 | IL51 | LVR51 | 231 |
| CC52 | IL52 | LVR52 | 232 |
| CC53 | IL53 | LVR53 | 233 |
| CC54 | IL54 | LVR54 | 234 |
| CC55 | IL55 | LVR55 | 250 |
| CC56 | IL56 | LVR56 | 251 |
| CC57 | N/A | LVR57 | 317 |
| CC58 | IL58 | LVR58 | 318 |
| CC59 | IL59 | LVR59 | 320 |
| CC60 | IL60 | LVR60 | 321 |
| CC61 | IL61 | LVR61 | 322 |
| CC62 | IL62 | LVR62 | 323 |
| CC63 | IL63 | LVR63 | 335 |

**Supplementary Table 1:** List of mouse Cecum, Ileum and Liver RNA samples used for RNAseq analysis.

| Depletion_A | Depletion_B | Pooled t test prob > \|t\| (assuming equal variances) | t test prob > \|t\| (assuming unequal variances) | Cohen's *d* | Significance |
|---|---|---|---|---|---|
| No Depletion | DP1_DPM | <0.0001 | <0.0001 | -6.477 | **** |
| No Depletion | DP1_DPM+2025 | <0.0001 | <0.0001 | -12.64 | **** |
| No Depletion | DP1_DPM+5050 | <0.0001 | <0.0001 | -9.14 | **** |
| DP1_DPM | DP1_DPM+2025 | 0.0061 | 0.0069 | -1.083 | ** |
| DP1_DPM | DP1_DPM+5050 | 0.0635 | 0.064 | -0.706 | ns |
| DP1_DPM+2025 | DP1_DPM+5050 | 0.3347 | 0.3353 | 0.358 | ns |

P > 0.05 = ns, P <= 0.05 = *, P <= 0.01 = **, P <= 0.001 ***, P <= 0.0001 ****

**Supplementary Table 2:** Results of two-tailed t-test of significance. One way analysis of % retained by depletion method (see Figure 3B). The comparison of DP1+DPM vs DP1+DPM+2025 is significant with a P < 0.01. To further study the magnitude of differences between groups, we used Cohen's *d* to calculate effect sizes. Effect sizes with absolute values greater than 0.8 (|*d*| > 0.8) indicate large differences between groups.

| BSSH App | Version | Settings | Purpose |
|---|---|---|---|
| FASTQ Toolkit | v2.2.5 | Subsample 20M FASTQ entries | Subsampling reads |
| Microbiome Metatranscriptomics | v1.0.1 | Default | Adapter and quality Trimming, rRNA read filtering, metatranscriptome aligments |
| DRAGEN Metagenomics Pipeline | v3.5.12 | Extended Kraken2 database, dehosting disabled | Taxonomic identification of rRNA reads |
| RNA Express | v1.1.0 | Default | Differential Gene Expression of mouse transcriptomes |

**Supplementary Table 3:** List of BSSH Apps used for RNAseq analysis
